# Selected research articles from the 2018 International Workshop on Computational Network Biology: Modeling, Analysis, and Control (CNB-MAC)

**DOI:** 10.1186/s12859-019-2830-5

**Published:** 2019-06-20

**Authors:** Byung-Jun Yoon, Xiaoning Qian, Tamer Kahveci, Ranadip Pal

**Affiliations:** 1 Department of Electrical and Computer Engineering, Texas A&M University, College Station, TX 77843-3128 USA; 2 TEES-AgriLife Center for Bioinformatics and Genomic Systems Engineering (CBGSE), College Station, TX 77845 USA; 3 Department of Computer and Information Science and Engineering, University of Florida, Gainesville, FL 32611-6125 USA; 4 Department of Electrical and Computer Engineering, Texas Tech University, Lubbock, TX 79409-3102 USA

## Introduction

The Fifth International Workshop on Computational Network Biology: Modeling, Analysis, and Control (CNB-MAC 2018) was held in Washington, D.C. on August 29, 2018. The workshop was organized in conjunction with the ACM Conference on Bioinformatics, Computational Biology, and Health Informatics (ACM-BCB), the flagship conference of the ACM SIGBio. The CNB-MAC workshop aims to provide an international scientific forum for presenting recent advances in computational network biology that involve modeling, analysis, and control of biological systems and system-oriented analysis of large-scale OMICS data.

CNB-MAC 2018 was co-chaired by Drs. Byung-Jun Yoon, Xiaoning Qian, Tamer Kahveci, and Ranadip Pal. The workshop featured 14 oral presentations and 9 poster presentations, which were selected by the workshop chairs based on the reviews performed by the technical committee members.

With the generous support provided by the National Science Foundation (NSF), Student Travel Grants have been awarded to student authors of outstanding research papers and posters that have been invited for presentation at CNB-MAC 2018. Dr. Ranadip Pal served as the award chair for CNB-MAC 2018. 13 awardees were selected by the award committee after a careful review of the applications and the submitted work.

## Research papers presented at CNB-MAC 2018

After the workshop, eleven papers [1–11] were accepted for publication in the CNB-MAC 2018 partner journals: BMC Bioinformatics and BMC Genomics. In the following we provide a brief summary of these selected papers.

Clinical studies often track dose-response curves of subjects over time which can be modeled separately in either time or dose without capturing the simultaneous evolution of the curves. Dhruba et al. [1] propose a parametric model to explain the dose-time response behavior and derive a recursive relation to predict dose-response curves over time for individuals using the corresponding dose-time proteomic data. By comparing the proposed recursive approach with individual dose-response predictive models at desired time points, Dhruba et al. [1] demonstrate that the recursive methodology provides a superior fit to the dose-time response behavior post drug application for both synthetic experimentation and pharmacological data from the HMS-LINCS database.

Missing values frequently arise in modern biomedical studies due to various reasons, including missing tests or complex profiling technologies for different omics measurements. Missing values can complicate the application of clustering algorithms, whose goal is to group data points based on some similarity criterion. In [2], Boluki et al. consider missing values in the context of optimal clustering, which finds an optimal clustering operator with reference to an underlying random labeled point process. They incorporate the missing value mechanism into the random labeled point process, and obtain the optimal clustering operator by marginalizing out the missing-value process. Optimal clustering with missing values obviates the need for imputation-based pre-processing of the given data, while at the same time possessing smaller clustering errors compared to various clustering approaches in comprehensive experimental studies on both synthetic and real-world RNA-seq data.

Constructing predictive models that can accurately differentiate the disease states for an individual based on microbiome profile can have significant impacts on precision medicine for many microbiome related diseases. Chieh Lo and Radu Marculescu propose a new framework MetaNN that utilizes neural networks to classify host phenotypes based on a new data augmentation method [3]. They show that MetaNN outperforms existing state-of-the-art models in terms of classification accuracy for both synthetic and real metagenomic data.

Polygenic diseases have phenotypes that are often difficult to genetically characterize, even with large-scale genome-wide studies. For example, both schizophrenia and autism have been associated with altered neuron motility, but the genetic underpinnings of this phenotype are not well understood. Bern et al. [4] formulate the computational problem of identifying candidate disease genes (e.g., schizophrenia) that are associated with a particular phenotype (e.g. aberrant cell motility) within a functional interaction network. They develop a semi-supervised learning approach to predict new candidates which outperforms peer methods across multiple gold standard datasets. The authors identify candidate motility regulation genes in schizophrenia and autism, selecting six schizophrenia candidates for follow-up experimental validation. This approach offers a framework for investigating biological processes that may be disrupted in polygenic diseases.

Elmansy and Koyutürk [5] elucidate the functional overlap between genomic markers of complex diseases that are identified on different populations. The authors use T2D as a model complex disease and develop a multi-layered framework, where genomic loci, protein-coding genes, biological pathways in which these proteins are involved, and networks of physical and functional interactions between these proteins are systematically evaluated for potential overlap. Results show that the overlap between different populations grow as the level of abstraction coarsens from genomic location to biological function. More interestingly, this study showed that differences in the biological processes that are implicated in different populations align with the targets of most commonly prescribed T2D drugs in each population. The authors also apply the proposed framework to genome-wide association studies on prostate cancer and observe similar results. The results support the notion that it can be useful to take ethnicity into account in making personalized treatment decisions for complex diseases.

Two temporal networks have co-evolving subnetworks if the evolving topologies of these subnetworks remain similar to each other as the network topology evolves over a period of time. In [6], Elhesha et al. consider the problem of identifying co-evolving subnetworks given a pair of temporal networks, which aim to capture the evolution of molecules and their interactions over time. Studying temporal networks in general and human aging specifically using Tempo enables us to identify aging related genes from those which are not tightly associated with aging related genes successfully. More importantly, Tempo takes the network alignment problem one huge step forward by moving beyond the classical static network models.

Hajiramezanali et al. [7] derive the optimal Bayesian classifier (OBC) for single-cell trajectories under potential model uncertainty. Partially-observed Boolean dynamical systems (POBDS) are used for modeling gene regulatory networks (GRN) to study noisy dynamic gene expression data of single cells. The application of the OBC becomes impractical for large GRNs, due to computational and memory requirements. To address this, the authors introduce a particle-based single-cell classification method to approximate OBC. The approximate method is highly scalable for large GRNs with demonstrated classification performance and significantly reduced complexity.

The hybrid stochastic simulation algorithm, proposed by Haseltine and Rawlings (HR), can significantly improve the efficiency of stochastic simulations for multiscale biochemical networks. Chen and Cao [8] investigate the negativity problem of the HR hybrid method, analyze and test it with several models including a realistic biological cell cycle system. Results show that usually the error caused by negative populations is negligible compared with approximation errors of the HR hybrid method itself, and sometimes negativity phenomena may even improve the accuracy. But for systems where negative species are involved in nonlinear reactions or some species are highly sensitive to negative species, the system stability will be influenced and may lead to system failure when using the HR hybrid method. For those circumstances, three remedies are proposed and studied for this negativity problem.

Enumerating all vertex-induced subgraphs in a single graph is an important problem in many application areas, including bioinformatics, computer vision, and optimization. Alokshiya et al. [9] propose a reverse-search algorithm for solving this problem by introducing a new parent-child relationship that guides the searching algorithm. The authors demonstrate that the proposed reverse search method outperforms existing algorithms on various random and chemical graphs with varying size and density. Moreover, the authors investigate the problem of integrating constraints defined on the similarities between the attributes of vertices in the reported subgraphs and propose efficient pruning strategies that dramatically reduce the search space and running time.

Identifying motifs in biological networks is a challenging task and provides valuable insights about the key functions of biological networks. The fact that biological interactions often depend on limited resource, which implies that network edges can participate in limited number of motifs simultaneously, further complicates the task. Ren et al. [10] develop a novel motif counting method, Partially Overlapping MOtif Counting (POMOC), that considers capacity levels for all interactions. Evaluation results on both synthetic and real datasets demonstrate that motif count using POMOC method significantly differs from existing motif count approaches. Application of this method to the *S. cerevisiae* transcriptional regulatory networks demonstrates that the POMOC method reveals topological differences of biological networks under different genetic backgrounds and experimental conditions.

Ahmadian et al. [11] investigate the effect of molecular noise and size control mechanism on the variabilities in cell cycle of the budding yeast ​*Saccharomyces cerevisiae*​. There are specific control mechanisms during cell cycle that maintain the cell size within a range from generation to generation. Such control mechanisms introduce substantial variabilities to important properties of the cell cycle such as growth and division. The proposed hybrid stochastic model provides an accurate, yet computationally efficient approach for simulation of an intricate system by integrating the deterministic and stochastic simulation schemes. The developed hybrid stochastic model can successfully capture several key features of the cell cycle observed in experimental data. In particular, the proposed model: 1) confirms that the majority of noise in size control stems from low copy numbers of transcripts in the G1 phase, 2) identifies the size and time regulation modules in the size control mechanism, and 3) conforms with phenotypes of early G1 mutants in exquisite detail.
